# Ultrasound morphology of inguinal lymph nodes may not herald an associated pathology

**DOI:** 10.1186/1756-9966-31-88

**Published:** 2012-10-18

**Authors:** Francesco M Solivetti, Fulvia Elia, Dario Graceffa, Aldo Di Carlo

**Affiliations:** 1 Radiology Department and Scientific Director, San Gallicano Dermatology Institute, Rome, Italy; 2 IRCCS Istituto Dermosifilopatico di Santa Maria e San Gallicano I.F.O, Via Elio Chianesi, 54, 00144, Rome, Italy

**Keywords:** Anatomical lymph nodes of the groin, Ultrasound, Melanoma, Cortical, Hilus

## Abstract

**Background:**

Among patients undergoing follow-up after surgery for melanoma, ultrasound (US) very often reveals lymph nodes in groin area, that do not show clear characters of a metastatic lesion yet that have atypical US features, which could result in diagnostic uncertainty. We evaluated such lesions among a cohort of patients.

**Methods:**

The study population consisted of patients who presented consecutively to our facility for a control between 1 January 2009 and 30 July 2010 and who had undergone surgery for a melanoma, at least 6 months earlier, in areas draining to lymph nodes of the groin but choosing – for this study - the opposite side to the natural drainage. The following parameters of the US performed on the lymph nodes were evaluated: number and size, aspects of the outline, including any extroflexion of the outline and contours morphology, homogeneity and thickness of the cortex and aspects of the hilus, characteristics of the vascularisation of the lymph node at color-power Doppler. A second US examination was performed on the same area after at least 12 months.

**Results and conclusions:**

We found a very high number of patients (42/124) with lymph nodes that did not appear to be fully normal at US examination, particularly those with structural alterations in the hilus and slight loss of physiologic curvature of the outlines, with moderate thickening of the cortex. Of the 124 patients, who were followed for at least one year, 42 showed these characteristics, and none of these showed any progression to malignancy at follow-up. Based on these results, we can conclude that focusing excessively on such US findings could lead to the inappropriate performance of additional diagnostic tests, with a consequent increase in management costs and a worsening of the quality of life for these patients.

## Background

In the follow-up of patients surgically treated for melanoma, ultrasound (US) examination of the naturally draining lymphatic stations very often reveals lymph nodes that appear as irregular or atypical, without clear aspects of metastases. These lymph nodes are particularly frequent in the groin area, even in healthy individuals, where they are often involved in acute or chronic micro-infections of the legs, caused by different noxae, such as trauma, micro-wounds, sports activities, incorrect epilation, or diabetes [1]. However, when a radiologist defines lymph nodes as “atypical” – even if typical metastatic patterns are not present - clinicians may be prone to perform additional yet unnecessary invasive procedures, such as agobiopsy or even excisional biopsy.

In light of these considerations, among individuals who had undergone surgery for melanoma, in areas theoretically draining to the groin lymph nodes, we assessed the occurrence of such lymph nodes and their US features (e.g., number, size, and morphologic and architectural characteristics), in order to better identify them.

Given that the US follow-up of groin lymph nodes consisted of bilateral examination of the stations in the inguinal area – as previewed by the Regional Health Service and for the unlikely occurrence of contralateral metastases - we decided to evaluate, for this study, the opposite side of theoretical lymphatic drainage of excised neoplasia.

According to the literature [2-7], to be defined as “normal”, a lymph node must be oval in shape, with a long-to-short-axis ratio (L/S ratio or roundness index) of > 2; it must have a regular and homogeneous central echoic hilus, a hypoechoic cortex with a homogeneous structure, with regular and well-defined outlines, without extroflexions, with vascular signals shown by color-power Doppler mainly located centrally and with a regular aspect, and scarce or absent peripheral vascular signals. The following ancillary findings with US are considered to be significant or potentially indicative of a pathology, although they have a low diagnostic value: lymph node diameter greater than 20 mm; thickness of the lymph node cortex greater than 2 mm, and an echo-poor central hilus.

## Materials and methods

The study population consisted of 150 patients who presented consecutively to our facility for a US evaluation of the groin lymph nodes between 1 January 2009 and 30 July 2010, and who had been surgically treated for melanoma in areas theoretically draining to the groin lymph nodes at least 6 months before the US control. At least one other US examination was performed at least 12 months after the first one.

The exclusion criteria were: drop out from the control visits; presence of metastatic lymph nodes; occurrence of other neoplastic lesions during the follow-up, including those of different histotype with respect to melanoma, in areas theoretically drained from the lymph nodal station being studied; a second surgical procedure in the same area; loco-regional dermatological or inflammatory pathologies (e.g., psoriasis, pemphigus etc) and pregnancy.

The characteristics of the study population are shown in Table 1.

**Table 1:** Characteristics of the study population

**Number of patients**	**124**
Sex	Males: 50; Females: 74
Age (in years, mean ± SD)	55.3 ± 13.81 (Min 12; Max 83)
Thickness of Superficial Spreading Melanoma (mm)	≥0.7; ≤1.3
Diabetes mellitus	8.06% of the sample population
Recent local trauma	9.67% of the sample population
Hair removal	38.71% of the sample population

*SD*: standard deviation.

A total of 124 individuals (74 females and 50 males) were included in the study; they ranged in age from 12 to 83 years (mean age of 55.3 years and modal age of 55.5 years).

The melanoma thickness, which we measured for descriptive purposes only according to the Breslow criteria, ranged from 0.7 to 1.3 mm.

We carefully chose the station contralateral to the site of the excised lesion and the sentinel node, to reduce the possibility of contamination from post-surgical interference and the statistical probability of metastases.

The same US apparatus was used for all patients (Esaotebiomedica Mylab 70XVG – Genova, Italia), and a 7.5-13 MHz linear array probe (type LA523) was adopted in all cases. All of the US examinations were performed by two expert radiologists (FMS and FE), who have, respectively, 35 and 12 years of experience in US activity and 12 and 6 years of experience in the field of dermatological oncology. The US examination was performed with the patient in a supine position, with the examined limb outwardly rotated and abducted, exercising sufficient pressure with the probe and, if necessary, varying the frequency based on the patient’s somatic habitus. We first performed a normal scan of the vascular axis and in all cases at least a second longitudinal scan, thus measuring two major orthogonal planes of the lymph node. The data were recorded on a previously developed form (Additional file 1: Attachment), and the images were recorded in our facility’s RIS-PACS system; if there were any doubts, the authors reviewed the data together to reach a consensus; if necessary, a third party was involved in reviewing the data. The lymph nodes that were not clearly metastatic but with non-typical aspects, were localized by dermographic pen on the corresponding skin surface and photographed digitally, so as to evaluate the same lymph node at the subsequent follow-up.

None of the lymph nodes showed US aspects that warranted additional diagnostic procedures other than follow-up controls.

The following US features of the lymph nodes were evaluated:

quantity and dimensions;

aspects of the outline;

homogeneity and thickness of the cortex, recording any extroflexion of the outline;

aspects of the hilus, in particular, disorganization;

color-power Doppler patterns of the vascularization.

We also recorded additional clinical data, in particular, the presence of diabetes mellitus, recent moderate loco-regional blunt traumas, habitual epilation of the lower limbs or pubic regions, and sports activities leading to frequent traumatic events. All data were recorded in a database (Microsoft Windows Excel, Microsoft Corp. Redmond, WA, USA), installed on a standard compatible IBM computer.

For the statistical analysis, we calculated the Spearman *r* index and performed unpaired Student’s *t* test; the level of significance was p < 0.05. The data are expressed as the mean ± standard deviation. The statistical analyses were performed using GraphPad Prism 5 software (GraphPad Software, Inc., La Jolla, CA – USA).

## Results

A total of 730 lymph nodes were observed, for a mean of 5.88 ± 2.009 per station and individual patient (range: 1-12). These data do not agree with the results of an anatomical study (8) in which the mean number of superficial and deep lymph nodes dissected at autopsy was 13.60 per side (range 5 -17). Regarding the size of the lymph nodes, the length of the major axis was as follows: < 10 mm for 168 lymph nodes, 10-20 mm for 490 lymph nodes, and > 20 mm for 72 lymph nodes; the latter represented 9.86% of all lymph nodes. The mean size of the largest lymph node in each patient in terms of the length of the major axis was 19.73 mm ± 6.294. Anatomically, the normal dimensions in terms of the maximum transverse diameter are usually between 1 and 2 cm [8]. According to a relatively recent study [9], which, however, used 10 MHz linear probes, most of the normal lymph nodes (181 out of 205) in the inguinal area had a maximum transverse diameter of 8 mm.

**Figure 1:**
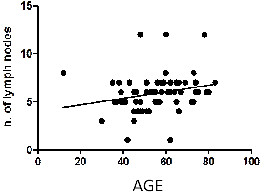
**Correlation between the size of the largest lymph node and age.** Spearman *r* 0.3172; 95% confidence interval 0.1440 to 0.4715; P value (two-tailed) 0.0003; P value summary ***.

**Figure 2:**
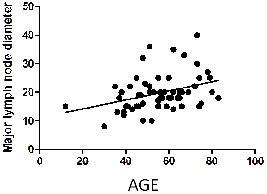
**Correlation between the size of the major lymph node diameter and age.** Spearman *r* 0.3475; 95% confidence interval 0.1772 to 0.4975; P value (two-tailed) <0.0001; P value summary ****.

The Spearman *r* index was 0.347 (p < 0.0001) for the statistical association between the number of lymph nodes per patient and age and 0.317 (p = 0.0003) for the association between the size of the largest lymph node and age (Figures 1 and 2); this finding is discussed in-depth below.

The mean cortical thickness was 1.277 ± 0.82 mm, which is basically consistent with the results of a study by Bedi, who reported that the normal maximum thickness was less than 3 mm [10]. In 14 (11.29%) of the 124 patients, we found that the cortical had irregular outlines (i.e., a mono-lobulated or multi-lobulated appearance). Moreover, none of the patients showed protrusions from the cortex into the soft tissue. In these 14 cases, the cortex consistently showed only slight focal thickening (< 4 mm, which only slightly exceeds normal thickness). Of these patients, 5 had a single extroflexion of the cortex; 6 patients had 2 and 3 patients had 3.

In 6 (4.84%) of the 124 patients, the cortex showed a structural irregularity; in particular, 3 of these patients showed macro-calcification and 3 showed hyperechoic areas.

The mean age of those patients with irregularities in the lymph nodes outlines and/or cortex was slightly higher than that of patients without these irregularities, though the difference was not statistical significant.

None of the patients had lymph nodes with marked focal alterations in vascularisation, yet cortical vascular signals were found in 3 of the 6 patients with cortical irregularities; these patients also showed extroflexions of the cortex exactly in correspondence with the color-power Doppler signal.

All patients showed fatty hilus, but 22 (17.74%) patients had at least one lymph node with a non-homogeneous or partially hypoechoic hilus. Although some recent studies have reported this pattern in non-pathological axillary and inguinal lymph nodes [11], according to other studies [3], these findings could be indicative of metastases.

With respect to the patient’s medical history, no associations were found between morphological anomalies in the lymph nodes and diabetes mellitus (reported by 10 of the 124 patients; 8.06%), recent moderate loco-regional trauma (12 patients, 9.67%), or habitual hair removal from the limbs and/or pubic region (48 patients, 38.71%).

Overall, the above results show that 42 (34%) of the 124 patients had at least one morphological alteration of lymph nodes that were considered to be potentially suspect for metastases, independently of the size of the lymph nodes. A size of > 2 cm, which was found in more than 20% of our patients, was not associated with the presence of irregular outlines or structural irregularities in the cortex. The characteristics of the lymph nodes are summarized in Table 2.

**Table 2:** Characteristics of the lymph nodes

**Number of lymph nodes detected**	**730; 5.88 ± 2,009/Patient/side**
Cortical thickness (Mean ± SD)	1.277 ± 0.82 mm
Cortical morphology alterations (cortical lobulation)	14/124 Patients (11.29% of the population)
Vascular alterations	0/124 Patients
Echo-poor or inhomogeneous central hilus	22/124 Patients (17.74% of the populations)

*SD*: standard deviation.

## Discussion

Based on the above results, which, however, refer to a relatively small cohort, the following considerations can be made:

US - with high frequency probes - is able to reveal only slightly fewer than 50% of the lymph nodes that are anatomically present in the lymph node stations studied. The dimensions of these lymph nodes are consistent with those found in the literature, where the maximum diameter reported is 1-2 cm [12]. However, in contrast to other studies, we report a relatively high percentage of lymph nodes (9.86%) with a maximum diameter that exceeds 2 cm.

These findings could indicate that lymph nodes that are large yet mainly fatty may be difficult to evaluate, especially using low frequency probes. This is because only the peripheral hypoechoic cortex has an adequate US contrast with the surrounding subcutaneous fatty tissue, and if this cortex is very thin, then detecting the lymph node can be very difficult. By contrast, lymph nodes that are smaller yet with a less fatty hilus would be theoretically easier to detect.

The mean thickness of the cortex in our study is consistent with the results of other studies [5,9,13] and basically consistent with anatomical data. However, based on the above hypothesis, it is possible that a thinner cortex would render the identification of such lymph nodes difficult, which would affect the percentage of lymph nodes with these characteristics.

A frequently observed anomaly (11.29% of the lymph nodes) was the extroflexion of the lymph node, which can be easily explained by physiological phenomena. In particular, the lymphatic vessels are afferent to the peripheral cortex, and the lymph, following filtration, exits from the hilus vessels. Thus it can be reasonably concluded that any response to “irritants”, whether inflammatory or neoplastic, would induce lymphocyte proliferation, which would be initially local, only later extending to within the lymph node. The irregularity of the outline, due to a local thickening of the cortex, appears to be related to an initial mild or moderate reaction to the irritating-inflammatory stimulus; it could also be the manifestation of a local outcome of past similar phenomenon.

We hypothesize that this alteration - frequently observed in non-neoplastic conditions - is reactive and non-specific; we can thus conclude that a higher number of extroflexions are unrelated to metastases, in that the malignant cells reach the lymph node from only a single or very few afferent lymphatic vessels, especially if the neoplasm is small. This hypothesis contradicts the findings of an another study, conducted at the axillary level, in which mono-lobulated and multi lobulated contours led to an increased relative risk of metastases (Odds Ratios of 2.1 and 3.8, respectively) [14]. Nonetheless, it is possible that in the previous studies [10,14] the focal thickening of the cortex was much greater than that in our study.

Structural irregularities of the cortex were present in fewer than 5% of the lymph nodes in our study and often consisted of macro-1calcification: theoretically, it can be reasonably speculated that this aspect could be related to inflammatory outcomes and does not seem to be indicative of metastases; a similar speculation can be formulated for the finding of hyperechoic areas within the cortex. It is obvious that greater problems could occur in cases with evidence of fluid areas, yet there are too few cases to draw any conclusions.

The structural alterations in the hilus are more difficult to explain. The pathological significance of these alterations has been extensively discussed, and the high percentage in our study (nearly 18% of lymph nodes) seems to indicate that these alterations are not indicative of an important pathology. This conclusion is conceptually logical if considering the physiopathology of the lymph node and its afferent vessels; however, further adequate autoptic studies of lymph nodes need to be performed.

Of interest is the finding that there were no significant vascular signals in the periphery of the lymph node, in particular, in the cortex. This finding, apart from the problems with the sensitivity of the instruments to slow flows, seems to indicate that the signals should be high to be indicative of a pathology. By contrast, moderate vascular signals appear to be physiopathologically compatible with an inflammatory or reactive condition, limited to the part of the cortex that coincides with the lymphatic vessels afferent to the irritated zone.

Surprisingly, we found no correlation between the size of the lymph nodes and diabetes or epilation, despite the fact that both of these conditions can act as irritants. The only important correlation was between age and the size of the lymph nodes, as if the various irritating phenomena that occur over time led, *ipso facto*, to a progressive increase in volume. However, age was not significantly associated with the presence of abnormalities in the outlines or the structure of the cortex, although empirically these should have the same significance.

In our opinion, the high incidence of patients with an anomaly in the structure of the lymph node that were negative at follow-up (34%; 42 of the 124 patients) demonstrate that certain US findings, especially the inhomogeneity of the hilus, the fibrotic areas in the cortex, and the moderate lobulation of the outlines, without important signals at color Doppler, are probably not indicative of a pathology. Moreover, needle aspirates and excisional biopsies often provide false-negative results in these cases [12]. The size of the lymph nodes does not seem to be indicative of a pathology, although there could be a coexisting low grade lymphoma, which could produce similar US findings.

## Conclusions

Based on these results, although the population studied was limited in size, there was a very high number of lymph nodes that were not indicative of significant pathologies, at least in the inguinal area, and for which US findings were the cause for concern or were even considered as suspect. This was especially the case for lymph nodes with structural alterations in the hilus and moderate extroflexions of the outline, with an associated moderate focal thickening of the cortex. The macro-calcifications, the areas of fibrosis and the presence of modest Doppler signals for the cortex appear to have little significance, at least with respect to metastases.

In conclusion, in the presence of the described anomalies (i.e., high number of lymph nodes, increased size, small lobulations of the outline, altered contour morphology, inhomogeneity or slight thickening of the cortex, anomalous hilus, and mild abnormal vascular pattern), we recommend clinical and US follow-up without additional invasive procedures, so as to avoid unnecessary stress to the patient and significant additional costs. However, an additional US control performed shortly after the first appears to be a reasonable and cost-effective solution, without running the risk of a poor prognosis because of initially unrecognized metastatic lesions.

## Competing interests

The authors declare that they have no competing interests.

## Authors’ contributions

FMS & FE: ultrasound; DG: statistical analysis; ADC: test revision. All authors read and approved the final manuscript.

## Supplementary Material

Additional file 1 **Attachment.** Protocol for inguinal lymph nodes: Patients undergoing follow-up for neoplastic pathologies for 1 year. Click here for file
