# Supplementary material for: Ultrasound morphology of inguinal lymph nodes may not herald an associated pathology
Source: J Exp Clin Cancer Res. 2012 Oct 18;31(1):88. doi: 10.1186/1756-9966-31-88 (PMC3551661; doi:10.1186/1756-9966-31-88)
Supplement: Additional file 1 — Attachment. Protocol for inguinal lymph nodes: Patients undergoing follow-up for neoplastic pathologies for 1 year. [file 1756-9966-31-88-S1.doc]

**Attachment**

Protocol for inguinal lymph nodes: Patients undergoing follow-up for neoplastic pathologies for 1 year

Patient name_________________________

Date of birth__________________________

Age___________Telephone______________

Original pathology______________________

Inguen opposite side of surgery

Total number of lymph nodes___________________

Number of lymph nodes:

smaller than 1 cm______ between 1 and 2 cm_______ larger than 2 cm_______

Size of largest lymph node (AXBXC)______________

Hilum recognised YES NO

Shape: Oval___ Round____ Other____

Irregular outlines YES NO

Homogeneous cortex YES NO

Mean thickness of cortex (mm)_____

Structural irregularities of cortex YES (type) NO

Vascularity: Regular___ Irregular___

Notes: Local inflammatory-infective pathologies

Diabetes YES NO

Hair removal / allergies YES NO

Traumas YES NO

Traumatic sports activities YES NO

Exclusion criteria: previous lymphnodal emptying (also on opposite side), active inflammatory pathologies, appearance of metastases
